# Psoriasin, one of several new proteins identified in nasal lavage fluid from allergic and non-allergic individuals using 2-dimensional gel electrophoresis and mass spectrometry

**DOI:** 10.1186/1465-9921-6-118

**Published:** 2005-10-19

**Authors:** Malin Bryborn, Mikael Adner, Lars-Olaf Cardell

**Affiliations:** 1 Laboratory of Clinical and Experimental Allergy, Department of Otorhinolaryngology, Malmo University Hospital, Lund University, Malmo, Sweden

## Abstract

**Background:**

Extravasation and luminal entry of plasma occurs continuously in the nose. This process is markedly facilitated in patients with symptomatic allergic rhinitis, resulting in an increased secretion of proteins. Identification of these proteins is an important step in the understanding of the pathological mechanisms in allergic diseases. DNA microarrays have recently made it possible to compare mRNA profiles of lavage fluids from healthy and diseased patients, whereas information on the protein level is still lacking.

**Methods:**

Nasal lavage fluid was collected from 11 patients with symptomatic allergic rhinitis and 11 healthy volunteers. 2-dimensional gel electrophoresis was used to separate proteins in the lavage fluids. Protein spots were picked from the gels and identified using mass spectrometry and database search. Selected proteins were confirmed with western blot.

**Results:**

61 spots were identified, of which 21 were separate proteins. 6 of these proteins (psoriasin, galectin-3, alpha enolase, intersectin-2, Wnt-2B and hypothetical protein MGC33648) had not previously been described in nasal lavage fluids. The levels of psoriasin were markedly down-regulated in allergic individuals. Prolactin-inducible protein was also found to be down-regulated, whereas different fragments of albumin together with Ig gamma 2 chain c region, transthyretin and splice isoform 1 of Wnt-2B were up-regulated among the allergic patients.

**Conclusion:**

The identification of proteins in nasal lavage fluid with 2-dimensional gelelectrophoresis in combination with mass spectrometry is a novel tool to profile protein expression in allergic rhinitis and it might prove useful in the hunt for new therapeutic targets or diagnostic markers for allergic diseases. Psoriasin is a potent chemotactic factor and its down-regulation during inflammation might be of importance for the outcome of the disease.

## Background

Increased vascular permeability and plasma exudation are important characteristics of allergic rhinitis leading to an increased amount of secreted proteins [1,2]. Earlier investigations with DNA microarray analysis [3] have described the gene expression in nasal mucosa. However, there is a considerable interest to identify some of the secreted proteins for a better understanding of the pathological processes and possibly to find new therapeutical targets or diagnostic markers for the disease.

Combining 2-dimensional gel electrophoresis (2-DE) with mass spectrometry (MS) have recently emerged as a method for identifying proteins in different biological samples. In short, proteins are separated in the first dimension according to their isoelectric points (pI) and then in the second dimension according to their molecular weight using SDS-PAGE. Each spot on the SDS-PAGE gel corresponds to one protein. The spots can be excised and further analysed and identified using mass spectrometry and database searching [4]. 2-DE together with MS has previously been used to investigate the protein content in nasal lavage fluid (NLF) [5] and a study of differences in the NLF protein content from smokers and non-smokers [6] is recently reported. However, changes in relation to allergic airway diseases have so far not been probed. The main purpose of this study was to use 2-DE in combination with MS and database search in order to map and identify the broad range of secreted proteins in NLF from individuals allergic to pollen (birch/timothy) and to compare that with NLF from non-allergic healthy individuals.

## Materials and methods

### Skin prick test

Skin prick tests (SPT) were performed with a standard panel of 10 common airborne allergens (ALK, Copenhagen, Denmark) including pollen (birch, timothy and artemisia), house dust mites (*D. Pteronyssimus *and *D. Farinae*), molds (*Cladosporium *and *Alternaria*) and animal allergens (cat, dog and horse). SPT were performed on the volar side of the forearm with saline buffer as negative and histamine chloride (10 mg/ml) as positive controls. The diameter of the wheal reactions were measured after 20 min with a ruler.

### Subjects

The study included 11 patients (6 women) with symptomatic birch and/or grass pollen induced intermittent allergic rhinitis and 11 healthy volunteers (7 women), serving as controls. The mean age of patients and controls was 43 (26–55) and 41 (24–55) years, respectively. The diagnosis of birch and/or grass pollen induced allergic rhinitis was based on a positive history of intermittent allergic rhinitis for at least 2 years and positive SPT to birch and/or grass. All patients were classified as having severe symptoms (itchy nose and eyes, sneezing, nasal secretion and nasal blockage) during pollen season and they had all been treated with antihistamines and nasal steroids during pollen seasons previous years. Patients had no continuous symptoms of asthma and they did not take any asthma medication. All patients presented a wheal reaction diameter >3 mm towards birch or timothy in SPT (roughly correponding to a 3+ or 4+ reaction when compared with histamine [7]). Exclusion criteria included a history of perennial symptoms, upper airway infection for the last 2 weeks before the time of visit and treatment with local or systemic corticosteroids during the last 2 months. The controls were all symptom-free, had no history of allergic rhinitis and had negative SPT to the standard panel of allergens as described above. They had no history of upper airway infection for 2 weeks before the time of visit and they were all free of medication. The study was approved by the Ethics Committee of the Medical Faculty, Lund University.

### Sample collection and preparation

Nasal lavage fluid was collected during either birch pollen (9 patients) or grass pollen season (2 patients). Patients were included when they had experienced substantial symptoms of rhinoconjunctivitis (itchy nose and eyes, sneezing, nasal secretion and nasal blockage) during at least 3 consecutive days. The majority of the patients were seen within 5–10 days after the first appearance of symptoms and a local pollen count.

Nasal lavage fluid was collected according to a previously described method [8]. After clearing of excess mucus from the nose sterile saline solution of room temperature was sprayed into both nostrils, respectively. The fluid was allowed to return passively and collected in a graded tube until 7 ml was recovered. NLFs were centrifuged at 1750 rpm at 4°C for 10 min to remove the cell content and the supernatants were stored at -70°C until sample preparation.

Before concentration of the samples NLFs were thawed and centrifuged at 12300 rpm at 4°C for 20 min to remove debris. Using Vivaspin 6 and Vivaspin 500 concentrators (Vivascience, Hannover, Germany) supernatants were concentrated and desalted. The protein concentration was determined using BCA Protein Assay Kit (Pierce Biotechnology, Rockford, USA) and resulted in a protein concentration of 1572–5625 μg/ml for healthy individuals and 1833–7867 μg/ml for allergic individuals. NLFs were stored at -70°C until analysed.

### 2-DE analysis

Samples were mixed with rehydration solution containing 8 M Urea, 2% CHAPS, 2.8 mg/ml DTT (Sigma-Aldrich, Steinheim, Germany), 0.5% IPG Buffer (pH 3–10) (Amersham Biosciences, Uppsala Sweden) and a small amount of bromophenol blue. For analytical gels 150 μg of protein was added to a final volume of 450 μl for each sample. For the preparative gels, one for healthy and one for allergic samples, 600 μg from a pool of samples was used. To be able to load as much as 600 μg on the preparative gels pooled samples were further concentrated using Microcon YM-3 (Millipore, Billerica, USA) before added to rehydration solution. Samples were incubated for approximately 15 min in room temperature in order to completely solubilize and denature the proteins. Samples were centrifuged at 13000 rpm for 10 min and thereafter loaded onto 24 cm 3–10 non linear IPG strips (Amersham Biosciences, Uppsala, Sweden). In-gel rehydration and isoelectric focusing (IEF) was performed over night (~60000 Vh) using Ettan IPGphor Isoelectric Focusing System (Amersham Biosciences, Uppsala, Sweden). After IEF strips were stored at -70°C until analysed. The IPG strips were equilibrated in SDS equilibration buffer (75 mM Tris, 6 M Urea, 30% glycerol, 2% SDS and 0.002% bromophenol blue (Sigma-Aldrich, Steinheim, Germany)) for 2 × 15 min. DTT (10 mg/ml) (Sigma-Aldrich, Steinheim, Germany) was added to the first and iodoacetamide (25 mg/ml) (Sigma- Aldrich, Steinheim, Germany) to the second equilibration step. After equilibration strips were loaded onto laboratory-made 12.5% acrylamide second dimension gels. SDS-PAGE was performed at constant effect (10 W/gel) for about 4 h and 30 min using the Ettan DALT II system (Amersham Biosciences).

### Staining of gels and gel image analysis

Second dimension gels were fixed in 30% ethanol and 10% acetic acid over night, washed 4 × 30 min in 20% ethanol and stained with the fluorescent dye ruthenium II tris-bathophenantroline disulfonate (1 μM) for about 6 h. Thereafter gels were destained in 40% ethanol and 10% acetic acid over night and washed with double distilled water for about 4 × 30–60 min [9]. All incubation and washing steps were performed with gentle agitation. Gels were kept dark in double distilled water at 4°C until scanned. The gels were automatically scanned using a robotic system together with a 9410 Typhoon scanner (488 nm laser) from Amersham Biosciences [10] and the gel images were analysed using the computer softwares Image master 2D Platinum (Amersham Biosciences) and Ludesi 2D Interpreter (Ludesi AB, Lund, Sweden). The volume in each spot was calculated as integrated optical density over the spot's area. The amount of protein in each spot was expressed as %VOL (ppm), that is the volume for the spot divided with the total volume for all spots in the gel.

### Spot picking, protein digestion and MALDI-TOF (Matrix Assisted Laser Desorption Ionization-Time Of Flight) analysis

Using the Ettan spot handling workstation (Amersham Biosciences) selected spots were automatically cut from the preparative gels, destained and enzymatically digested with trypsin (porcine Sequencing Grade Modified Trypsin, Promega, Madison, USA). The tryptic peptides were then spotted onto a MALDI target plate [11]. The MALDI target plates were loaded in a Micromass M@ldi MALDI-TOF mass spectrometer (Waters, Milford, USA) for analysis of the peptide masses.

### Database search

Peptide masses retrieved from MALDI-TOF analysis spectra were submitted to a database (IPI human 1.38) [12] by using the search engine PIUMS [13]. The following matcher parameters were used: constant modification of cysteine by carbamidomethylation, variable modification of methionine by oxidation and maximum 1 missed cleavage for trypsin. A protein hit was considered significant if the PIUMS quality score was ≥ 4.7, which corresponds to an expectation value of 0.01. A search in IPI human 1.38 was also done using the search engine Mascot and the results from this search were compared with the results from PIUMS.

### Western blot

NLFs were mixed with SDS sample buffer, heated at 95–100°C for 5 min and centrifuged at 10 000 rpm for 10 min. Equal amounts of the samples were loaded onto NuPAGE Bis-Tris 4–12% gel (Invitrogen, Carlsbad, USA), separated by electrophoresis (Mini vertical gel system, Thermo EC, Waltham, USA), and blotted to Immobilon-P PVDF membranes (Millipore, Billerica, USA). Membranes were blocked in buffer 1 (Tris-HCl 10 mM pH 7.4, NaCl 0.9% and dry milk 5%) and then incubated overnight with primary antibody (1 μg/ml) against psoriasin, galectin-3 (Abcam, Cambridge, UK), Wnt-2B (Zymed, South San Francisco, USA) and alpha enolase (Santa Cruz, Santa Cruz, USA), respectively. Membranes were washed 2 times with buffer 1 followed by incubation for approximately 2 h with HRP conjugated secondary antibody (50 ng/ml). After 2 washes with buffer 2 (Tris-HCl 10 mM pH 7.4, NaCl 0.9% and Tween 20 0.05%) membranes were incubated for 5 min in SuperSignal West Pico solution (Pierce Biotechnology, Rockford, USA). The chemiluminescence was detected using MAN-X X-ray system (Fujifilm Science Imaging systems, USA). Developed films for quantitative analysis were scanned and analysed in ImageQuant (Molecular dynamics, Sunnyvale, USA). There were no antibodies available against hypothetical protein MGC33648 and the relevant fragment of intersectin-2. Hence, these proteins could not be assessed with western blot.

### Statistical analysis

All values were expressed as mean values ± SEM. Statistical analysis of the protein expression was performed in Ludesi 2D interpreter (Ludesi AB) using one-way ANOVA.

## Results

### Novel proteins in nasal lavage fluid

**Figure 1:**
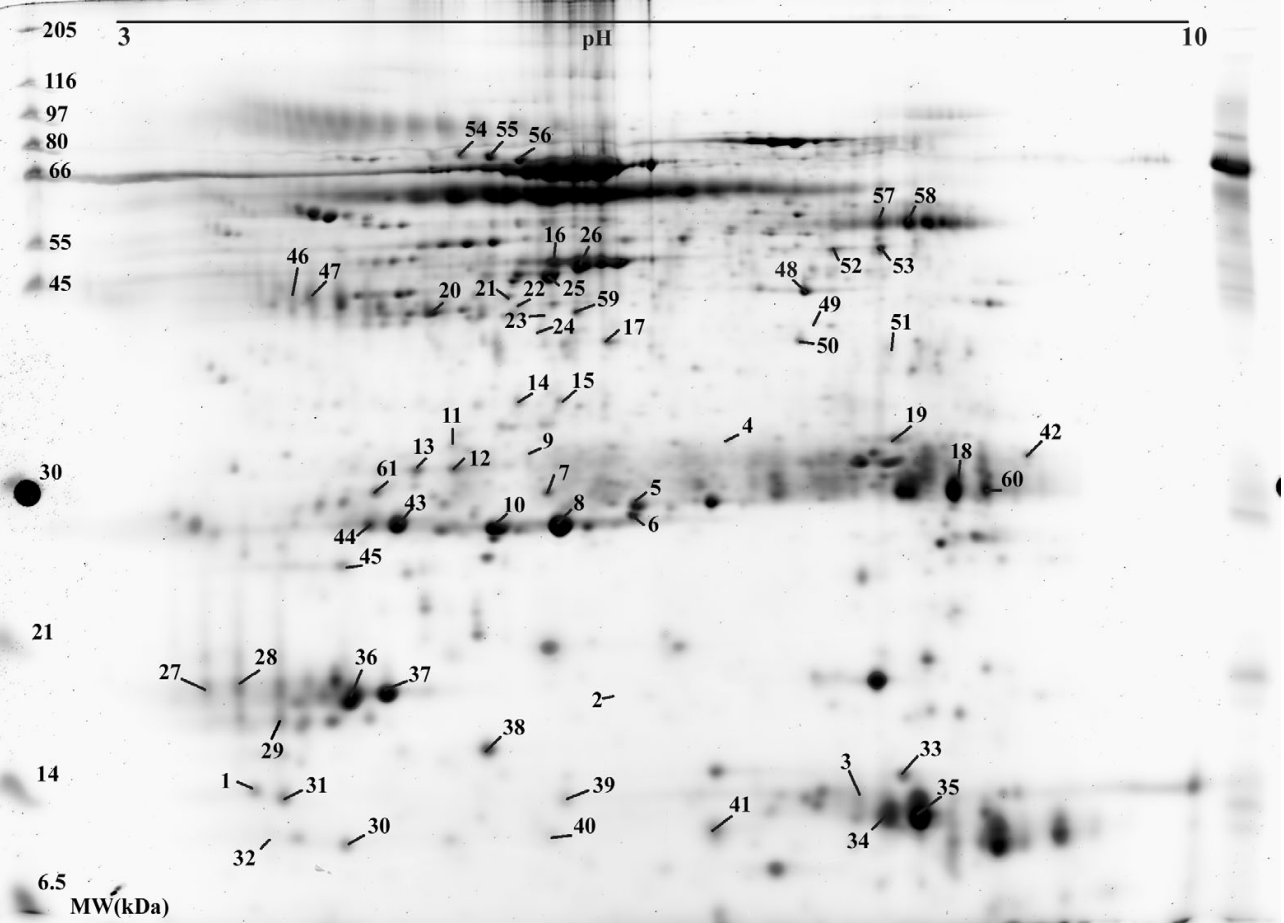
2-DE protein pattern for NLF from a healthy non-allergic individual. The protein name for each numbered spot is presented in table 1.

**Figure 2:**
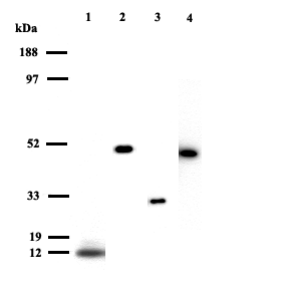
Western blot analysis of NLF from healthy non-allergic individuals. (1) Psoriasin, (2) Wnt-2B, (3) Galectin-3, (4) Enolase.

Of the spots picked from the 2D-gels and submitted to MALDI-TOF analysis 61 spots were identified (figure 1 and table 1). 21 of these were identified as separate proteins. The majority of the proteins has previously been identified in NLF [5,6,14] but this is the first study where psoriasin, galectin-3, alpha enolase, intersectin-2, Wnt-2B and hypothetical protein MGC33648 have been recognized. The occurrence of psoriasin, galectin-3, Wnt-2B and alpha enolase was confirmed with western blot (figure 2).

**Table 1:** Identified proteins in nasal lavage fluid from allergic and non-allergic individuals.

**Gel no.**	**Protein**	**Accession no. (Swissprot/IPI)**	**MW (kDa) (theoretical)**	**pI (theoretical)**
1	Albumin	P02768	69.4	5.9
2	Albumin	P02768	69.4	5.9
3	Albumin	P02768	69.4	5.9
4	Albumin	P02768	69.4	5.9
5	Albumin	P02768	69.4	5.9
6	Albumin	P02768	69.4	5.9
7	Albumin	P02768	69.4	5.9
8	Albumin	P02768	69.4	5.9
9	Albumin	P02768	69.4	5.9
10	Albumin	P02768	69.4	5.9
11	Albumin	P02768	69.4	5.9
12	Albumin	P02768	69.4	5.9
13	Albumin	P02768	69.4	5.9
14	Albumin	P02768	69.4	5.9
15	Albumin	P02768	69.4	5.9
16	Albumin	P02768	69.4	5.9
17	Albumin	P02768	69.4	5.9
18	Albumin	IPI00216773	45.2	6.0
19	Albumin	IPI00384697	47.4	6.3
20	Albumin	IPI00384697	47.4	6.3
21	Albumin	IPI00384697	47.4	6.3
22	Albumin	IPI00384697	47.4	6.3
23	Albumin	IPI00384697	47.4	6.3
24	Albumin	IPI00384697	47.4	6.3
25	Albumin	IPI00384697	47.4	6.3
26	Albumin	IPI00384697	47.4	6.3
27	Prolactin-inducible protein	P12273	16.6	8.3
28	Prolactin-inducible protein	P12273	16.6	8.3
29	Prolactin-inducible protein	P12273	16.6	8.3
30	Prolactin-inducible protein	P12273	16.6	8.3
31	Cystatin S	P01036	16.2	4.9
32	Cystatin S	P01036	16.2	4.9
33	Cystatin SN	P01037	18.8	6.8
34	Hemoglobin beta chain	P68871	16.0	7.3
35	Hemoglobin beta chain	P68871	16.0	7.3
36	**Intersectin 2,** **(splice isoform 2)**	Q9NZM3-2	190.5	8.4
37	Lipocalin-1	P31025	19.3	5.4
38	Transthyretin	P02766	15.9	5.5
39	Calgranulin B	P06702	13.2	5.7
40	**Psoriasin (S100A7)**	P31151	11.5	6.3
41	**Psoriasin (S100A7)**	P31151	11.5	6.3
42	**Galectin-3**	P17931	26.2	8.6
43	Apolipoprotein A1	P02647	30.8	5.5
44	Apolipoprotein A1	P02647	30.8	5.5
45	Apolipoprotein A1	P02647	30.8	5.5
46	Alpha-2 glycoprotein 1, zink	P25311	34.3	5.9
47	Alpha-2 glycoprotein 1, zink	P25311	34.3	5.9
48	Serotransferrin	P02787	77.1	6.8
49	Serotransferrin	P02787	77.1	6.8
50	Serotransferrin	P02787	77.1	6.8
51	Serotransferrin	P02787	77.1	6.8
52	**Alpha enolase**	P06733	47.0	7.0
53	**Alpha enolase**	P06733	47.0	7.0
54	Hemopexin	P02790	51.7	6.5
55	Hemopexin	P02790	51.7	6.5
56	Hemopexin	P02790	51.7	6.5
57	Ig gamma 2 chain c region	P01859	35.9	7.7
58	**Wnt-2B protein** **(splice isoform 1)**	Q93097-1	43.8	9.3
59	Fibrinogen beta chain	P02675	55.9	8.5
60	**Hypothetical protein MGC33648**	IPI00168581	34.2	9.0
61	Actin, cytoplasmic 1 or 2	P60709	41.7	5.3

Proteins in bold are newly identified in NLF.

### Differences in protein expression between allergic and non-allergic individuals

**Figure 3:**
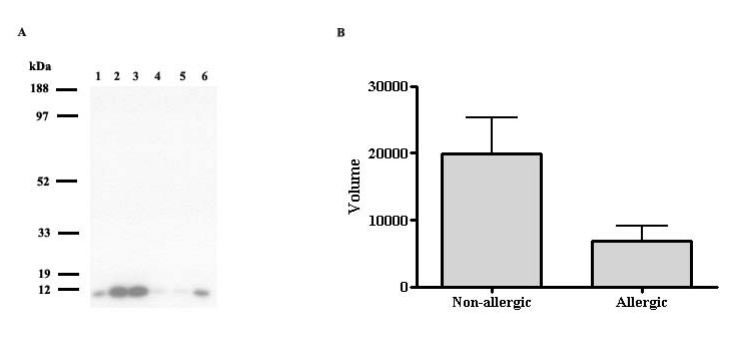
Expression analysis of psoriasin with western blot. A: Western blot analysis of NLF from three healthy non-allergic individuals (1–3) and three allergic individuals (4–6) demonstrating the levels of psoriasin. 1 μg of total protein was loaded in each lane. B: Quantitative analysis of western blot with ImageQuant (Molecular Dynamics, USA). Each data point represents the mean ± SEM.

14 spots exhibited a clear difference in the protein content when the material from allergic and non-allergic individuals was compared (table 2). 8 of these spots were identified as different fragments of albumin and all these were up-regulated in allergic individuals (1.8- to 2.6-fold). Wnt-2B (splice isoform 1), transthyretin and Ig gamma-2 chain c region were also found to be up-regulated in allergic compared to non-allergic individuals (2.5-, 1.6- and 2.1-fold, respectively). In contrast, prolactin-inducible protein and two forms of psoriasin were found to be down-regulated in allergic individuals (2.0-, 2.0- and 3.4-fold, respectively). The psoriasin levels in nasal lavage fluids from three patients with allergic rhinitis and three controls were also assessed using western blot analysis (figure 3A). Quantitative analysis revealed reduced levels among the allergic patients; 19962 ± 5410 for the non-allergic individuals compared to 6834 ± 2258 for the allergic individuals (figure 3B).

**Table 2:** Proteins differently expressed in allergic and non-allergic individuals.

**Protein**	**No.**	**Non-allergic**^a^	**Allergic**^a^	**Fold changes**
Psoriasin	40	1831 ± 425	543 ± 110*	-3.4
Psoriasin	41	7410 ± 1675	3689 ± 650*	-2.0
Prolactin-inducible protein	29	2322 ± 491	1130 ± 194*	-2.0
Transthyretin	38	1182 ± 177	1902 ± 286*	1.6
Ig gamma 2 chain c region	57	3493 ± 590	7349 ± 1137*	2.1
Wnt-2B protein (splice isoform 1)	58	3360 ± 668	8405 ± 1761*	2.5
Albumin fragment	6	803 ± 210	1580 ± 258*	2.0
Albumin fragment	11	363 ± 83	957 ± 247*	2.6
Albumin fragment	15	386 ± 99	606 ± 116*	2.4
Albumin fragment	17	514 ± 115	927 ± 86*	1.8
Albumin fragment	21	407 ± 74	1008 ± 183*	2.5
Albumin fragment	23	139 ± 50	324 ± 72*	2.3
Albumin fragment	20	2135 ± 374	3991 ± 638*	1.9
Albumin fragment	4	219 ± 54	500 ± 92*	2.3

^a ^The amount of protein is expressed as mean %VOL (ppm) ± SEM.

* p < 0.05

## Discussion

The present study is the first where 2-DE in combination with MS has been used to study differences between allergic and non-allergic individuals. It reveals the presence of six novel NLF proteins: psoriasin, galectin-3, alpha enolase, intersectin-2, Wnt-2B and hypothetical protein MGC33648. One of these novel proteins, psoriasin, was markedly down-regulated in allergic individuals. The same was found for prolactin-inducible protein, whereas different fragments of albumin together with Ig gamma 2 chain c region, transthyretin and splice isoform 1 of Wnt-2B were up-regulated among the allergic patients.

Increased vascular permeability and plasma exudation are important characteristics of allergic rhinitis resulting in an increased secretion of proteins. Several of the secreted proteins are collected in NLF and their identification is of importance for understanding pathological mechanisms in allergic rhinitis. DNA microarray technology is a relatively new method for analysing gene expression in different samples and it has recently been used in allergy research [15,16]. With DNA microarray technology it is possible to map genes that are up- or down-regulated in tissues or cells involved in allergic disease, something that might contribute to the identification of new pathological mechanisms or therapeutic targets [17]. However, all regulatory mechanisms are not operated at the transcriptional level. Hence, one of the disadvantages with DNA microarray technology is that the detected mRNA levels not always correlate with the actual protein levels in the sample. 2-DE together with MS-analysis is a powerful method to profile the protein expression in different samples. Two previous studies have used this methodology to analyse proteins in lavage fluids from the upper airways [6,14]. The present data now demonstrate that this approach also can be used to compare healthy and pathological samples in order to get an overview of which proteins that can be of importance for the development of allergic diseases.

Most of the 21 proteins identified in the present study correlate well with proteins found in NLF from normal, healthy individuals in previous studies [5,6,14]. However, 6 of the proteins (psoriasin, galectin-3, alpha enolase, intersectin-2, Wnt-2B and hypothetical protein MGC33648) have not previously been described in NLF. Psoriasin, also called S100A7, belongs to the S100 protein family and like other members in this family (for example calgranulin B) it has calcium-binding properties. It was first identified in psoriatic skin [18] where it is highly up-regulated. Psoriasin is thought to be involved in inflammation since it is a potent chemotactic factor for CD4+ T lymphocytes and neutrophils [19]. Galectin-3 belongs to a family of β-galactoside-binding animal lectins [20]. It is expressed in mast cells, monocytes/macrophages, neutrophils and eosinophils. Although galectin-3 lacks signal peptide, it can be secreted [21] and it functions as chemotactic factor for monocytes and macrophages [22]. Galectin-3 also has the ability to bind Ig-E and increased mRNA levels for galectin-3 have been found in neutrophils derived from the blood of allergic patients [23]. Alpha enolase is a ubiquitous multifunctional enzyme involved in many different processes [24]. It has been reported as an important allergen in inhalant allergies to fungi [25] and specific IgE antibodies have been found in patients allergic to fungi [26], all corroborating the notion that alpha enolase might play a role in allergic reactions. One spot was identified as splice isoform 2 of intersectin-2, a cytoplasmic protein involved in endocytosis [27]. The spot identified is probably a degradation product of intersectin-2 since it is present at a much lower MW than the theoretical value. Wnt-2B is a developmental protein that might play a role as hematopoietic growth factor [28].

The role of these newly identified proteins in allergic rhinitis is not known and they all render further investigation. However, special attention might be drawn to the two different forms of psoriasin [29] found to be down-regulated during allergic rhinitis in the present study. Since allergic rhinitis is an inflammatory disease and psoriasin is a chemotactic factor for inflammatory cells one could have expected the opposite. One explanation for this reversed condition is that psoriasin in addition to its chemotactic properties has an other not yet discovered role in the inflammation process. In this context, it is also essential to recognize that inflammation is normally a self-resolving process with the existence of both positive and negative regulators that ultimately allow complete resolution and homeostasis. In the absence of resolution and clearance or in the event of a dampened healing response, persistent inflammation can arise in the form of tissue damage as associated with chronic disease. Thus, the down-regulation of psoriasin during the allergic inflammation could be of importance for the natural resolution of the disease.

Previous findings [23] have suggested that galectin-3 is involved in the inflammatory reaction seen in allergic patients. However, in the present study no differences in the galectin-3 content were seen when material from allergic and non-allergic individuals were compared. Alpha enolase was identified in two spots but any quantitative difference between allergic and non-allergic individuals could not be detected. In contrast, splice isoform 1 of Wnt-2B was found to be up-regulated (2.5-fold) among allergic individuals. Such an increase of the Wnt-2B secretion might be related to the increased growth and maturation stimulation of eosinophils and neutrophils often seen during the allergic inflammation.

In addition to the novel NLF proteins psoriasin and Wnt-2B, a group of other proteins were also found to be differently expressed during the allergic inflammation. There was a 2.0-fold decrease of one form of prolactin-inducible protein (PIP) in allergic individuals. PIP is expressed in exocrine organs like sweat, salivary and lacrimal glands [30]. The functions of PIP is not completely known but it has CD4-binding properties and is a strong inhibitor of T lymphocyte apoptosis [31]. A down-regulation of PIP might therefore be associated with an increased apoptosis of T lymphocytes, something that might contribute to a limitation of the inflammatory process. The theoretical pI for PIP is 8.3 but it was detected in the gel at a pI around 4–5. Without its signal peptide the theoretical pI decreases to 5.4 which is closer to our observation. Transthyretin, also called prealbumin, is a plasma protein involved in the transport of thyroxine and retinol [32]. The small up-regulation of transthyretin detected in allergic individuals (1.6-fold) is probably due to the increased plasma exudation seen in allergic rhinitis [2].

Several of the spots were identified as the same protein. Hence, many proteins are present in different forms. The different forms may result from post-translational modifications like phosphorylation, glycosylation, acetylation or degradation of the proteins. All spots identified as albumin are probably different forms and fragments of albumin and since albumin is highly abundant in plasma this high amount of degradation products in NLF is expected. It is not surprising that a few of these fragments were up-regulated in allergic individuals since this only confirms previous findings that the secretion of albumin is increased in allergic individuals. Ig gamma 2 chain c region was also found to be up-regulated in allergic individuals which also confirms previous findings [33].

## Conclusion

2-DE in combination with MS-analysis appears to be a powerful method to profile the protein expression and compare healthy samples with pathological samples. In this study both previously identified and newly identified proteins were detected in NLF using this method. Some of these proteins, like psoriasin, Wnt-2B and PIP were found to be differently expressed in allergic and non-allergic individuals. Further investigations are needed to explain the pathological significance of these proteins. It is possible that some of them can be defined as new therapeutic targets or diagnostic markers for allergic diseases.

## Competing interests

The author(s) declare that they have no competing interests.

## Authors' contributions

MB performed the sample preparation, 2-DE, gel image analysis, analysis of MALDI results, database search, western blot and drafted the manuscript. MA and LOC conceived the study, participated in its design and coordination and helped to draft the manuscript.
